# Collagen‐rich deposit formation in the sciatic nerve after injury and surgical repair: A study of collagen‐producing cells in a rabbit model

**DOI:** 10.1002/brb3.1802

**Published:** 2020-08-15

**Authors:** Jolanta Fertala, Michael Rivlin, Mark L. Wang, Pedro K. Beredjiklian, Andrzej Steplewski, Andrzej Fertala

**Affiliations:** ^1^ Department of Orthopaedic Surgery Sidney Kimmel Medical College Thomas Jefferson University Philadelphia PA USA; ^2^ Rothman Institute of Orthopaedics Thomas Jefferson University Hospital Philadelphia PA USA

**Keywords:** axons, collagen, fibrosis, myofibroblasts, nerve regeneration, neural scar, peripheral nerve injuries

## Abstract

**Introduction:**

Posttraumatic scarring of peripheral nerves produces unwanted adhesions that block axonal growth. In the context of surgical nerve repair, the organization of the scar tissue adjacent to conduits used to span the gap between the stumps of transected nerves is poorly understood. The goal of this study was to elucidate the patterns of distribution of collagen‐rich scar tissue and analyze the spatial organization of cells that produce fibrotic deposits around and within the conduit's lumen.

**Methods:**

Employing a rabbit model of sciatic nerve transection injury, we studied the formation of collagen‐rich scar tissue both inside and outside conduits used to bridge the injury sites. Utilizing quantitative immunohistology and Fourier‐transform infrared spectroscopy methods, we measured cellular and structural elements present in the extraneural and the intraneural scar of the proximal and distal nerve fragments.

**Results:**

Analysis of cells producing collagen‐rich deposits revealed that alpha‐smooth muscle actin‐positive myofibroblasts were only present in the margins of the stumps. In contrast, heat shock protein 47‐positive fibroblasts actively producing collagenous proteins were abundant within the entire scar tissue. The most prominent site of transected sciatic nerves with the highest number of cells actively producing collagen‐rich scar was the proximal stump.

**Conclusion:**

Our findings suggest the proximal region of the injury site plays a prominent role in pro‐fibrotic processes associated with the formation of collagen‐rich deposits. Moreover, they show that the role of canonical myofibroblasts in peripheral nerve regeneration is limited to wound contracture and that a distinct population of fibroblastic cells produce the collagenous proteins that form scar tissue. As scarring after nerve injury remains a clinical problem with poor outcomes due to incomplete nerve recovery, further elucidation of the cellular and spatial aspects of neural fibrosis will lead to more targeted treatments in the clinical setting.

## Graphical abstract

In this study, we analyzed collagen‐rich deposits formed in response to the injury of the sciatic nerve. Utilizing microscopic and spectroscopic techniques, we determined the distribution and architecture of fibrotic deposits formed in the proximal and the distal stumps of the injured nerve.

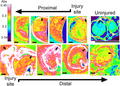

## 1 INTRODUCTION

Peripheral nerve injury is a significant medical problem that affects about 3% of patients with trauma to extremities (Noble, Munro, Prasad, & Midha, 1998). Although peripheral nerves have the intrinsic ability to regenerate, neural scar formation often hampers the nerve repair process (Ngeow, 2010). One of the key steps in nerve regeneration is the Wallerian degeneration of the distal axon segment. During this process, Schwann cells and macrophages actively proliferate and phagocytose myelin and axonal debris (Hall, 2005; Toews, Barrett, & Morell, 1998; Wagner & Myers, 1996). In the course of regeneration, acutely denervated Schwann cells decrease the production of myelin‐associated proteins. Subsequently, the Schwann cells present within the distal stump and the tip of the proximal stump start to proliferate. The de‐differentiated daughter cells start to produce regeneration‐associated proteins that promote axonal elongation.

Neural scar formation is a part of the regenerative process, but the excessive buildup of extraneural and intraneural scar tissue hampers this process significantly. The extraneural scars may form adhesions with surrounding tissues, thereby impairing local sliding of the injured nerve in response to the movement of a limb or a digit (Dilley, Lynn, Greening, & DeLeon, 2003). As a result, these adhesions may increase the intraneural tension and compromise the nerve repair processes (Millesi, Zoch, & Rath, 1990; Millesi, Zoch, & Reihsner, 1995; Smith, Wolf, Lusardi, Lee, & Meaney, 1999). The intraneural scars block, deflect, or delay the growing axons from traversing through the injury sites. Furthermore, the extraneural and intraneural scarring may also compromise the microvascular bed of a nerve, which may lead to secondary axonal degeneration (Hall, 2005).

In addition to the axonal growth, regeneration of the extracellular matrix (ECM) is a crucial element of the repair of injured peripheral nerves. Among elements of the ECM, collagenous proteins play a crucial role by participating in the formation of neural basement membranes and contributing to the mechanical stability of the nerves. In addition to playing a physiological role, collagenous proteins, most notably fibrillar collagen I and collagen III, participate in the formation of unwanted neural scars (Chen, Cescon, & Bonaldo, 2015; Salonen, Lehto, Vaheri, Aro, & Peltonen, 1985).

Integral to the biosynthesis of functional collagen molecules is protein chaperones that control the formation of the collagen triple helix. In particular, heat shock protein 47 (HSP47), a collagen‐specific protein chaperone that is upregulated during scar formation in a variety of human tissues, including injured joint capsules and nerves, plays a key role. Because of its close association with the collagen production, HSP47 serves as a reliable marker of collagen‐producing cells (Rivlin et al., 2017; Steplewski et al., 2016).

The origin of cells that produce collagen‐rich neural scars is not well established. While studies have documented how Schwann cells contribute to the production of fibril‐forming collagens, the origin of the fibroblastic cells is not clear (Lorimier et al., 1992). To monitor the proliferation of injury‐activated fibroblasts, scientists often measure alpha‐smooth muscle actin (αSMA), a marker for myofibroblasts, upregulated during the fibrotic processes (Tomasek, Gabbiani, Hinz, Chaponnier, & Brown, 2002). In uninjured peripheral nerves, αSMA is present in pericytes surrounding blood vessels and within a layer of cells forming the perineurium (Joseph et al., 2004). In injured peripheral nerves, however, the distribution of αSMA‐positive fibroblastic cells is less understood.

While collagen‐rich tissue formed during the few weeks after injury maintains the continuity of healed nerves, protecting the fragile site of the nerve damage right after trauma is a challenging problem. To maintain the structural integrity of injured nerves during the healing process, surgeons apply various coaptation techniques to bring together the proximal and the distal stumps. These techniques include suturing the stumps, fusing them using laser welding, and applying fibrin glue to reinforce the repair sites (Menovsky & Beek, 2001). Although, in certain cases, it is possible to connect the proximal and distal stumps without creating any unwanted tension, more substantial defects require bridging the gap. The gold standard for bridging the neural defect is nerve autografts. When harvesting autografts is not possible or suboptimal, surgeons employ various artificial conduits or nerve connectors to bridge the gaps between the proximal and the distal stumps of injured nerves (Houshyar, Bhattacharyya, & Shanks, 2019; Ray & Mackinnon, 2010). An ideal conduit exhibits mechanical support for the nerve, isolates the injury site from scar tissue invasion, and serves as a guide for regenerating axons (Houshyar et al., 2019).

Some studies demonstrate that applying conduits to repair the peripheral nerves improves the outcomes. A systemic review of the outcomes of nerve repair surgeries indicated that using nerve conduits was beneficial compared with standard suture repair when the nerve gap was 4 mm or less; however, there was no significant difference when the gap was 5–7 mm (Narayan, Arumugam, & Chittoria, 2019). Furthermore, the authors noted that because surgeons use different conduit materials and apply suturing techniques that cause varying tensions, the true value of the conduits for nerve regeneration is difficult to assess conclusively (Narayan et al., 2019).

Researchers also report significant limitations of using the conduits. Crucial limitations include premature deterioration of conduit materials, creation of unwanted acidic environment, and tissue necrosis (Riccio, Marchesini, Pugliese, & De Francesco, 2019). Moreover, researchers found that collagen‐based conduits improved outcomes in only 43% of patients, suggesting that the utility of these conduits may be limited by the inflammatory response that leads to fibrotic scarring of the nerves (Wangensteen & Kalliainen, 2010). The noncollagenous materials used to produce conduits have limitations too. In contrast to collagen‐based constructs, silicone conduits attract contractile myofibroblasts, which may impact the composition of the cell pool associated with wound healing (Chamberlain, Yannas, Hsu, & Spector, 2000). Despite these limitations, conduits remain an attractive tool to repair injured nerves due to their ability to guide regenerating axons and accumulate neurotrophic factors.

Unlike directly suturing the proximal and the distant stumps, using a conduit compartmentalizes the sites of neural injury into the intraluminal domain and the extraluminal space. Because of the conduit barrier, these distinct spaces maintain unique biological properties and mechanical characteristics (Chamberlain et al., 2000). In an effort to gain further insight into formation of the scar tissue within the luminal and the extraluminal domains, we employed a rabbit model of the sciatic nerve injury to investigate the fibrillar architecture of the scar tissue and the distribution of cells that build the scar deposits. In doing so, our study sheds new light on mechanisms of regeneration of the peripheral nerves following surgical repair.

## 2 MATERIALS AND METHODS

### 2.1 Institutional approval of animal studies

The Institutional Animal Care and Use Committee of Thomas Jefferson University approved all animal studies presented here.

### 2.2 Ethics approval

The authors declare that they complied with the guidelines for the care and use of laboratory animals as described by the U.S. National Institutes of Health.

### 2.3 Nerve injury model

Applying a muscle‐sparing approach, a surgeon accessed the proximal sciatic nerve of the right legs of 12‐month‐old White New Zealand female rabbits placed under general anesthesia (Miller et al., 2018). After transecting the nerve, the ends of the proximal and the distal stumps were pulled into a conduit (Axoguard Nerve Connector; Axogen Corp.) and secured on each end with sutures, then sealed with the fibrin glue (TISSEEL; Baxter International Inc.). Following surgery, the rabbits were allowed free cage activity. Subsequently, the rabbits were sacrificed at 2 weeks (*n* = 3), 4 weeks (*n* = 3), 6 weeks (*n* = 4), and 10 weeks (*n* = 4) after injury, and then, the corresponding segments of the injured and uninjured nerves were harvested for histological and spectroscopic analyses.

### 2.4 Processing nerve tissue

Our study mainly focused on the group of rabbits sacrificed 4 weeks after injury. At this time point, the Wallerian degenerative process nears completion, the scar tissue is well‐developed, and regenerating nerve fibers start to appear (Abe, Doi, & Kawai, 2005; Yamasaki et al., 2015). Additionally, nerves collected from the supplementary groups were utilized for further morphological analysis (see below).

After dissecting the sciatic nerve segment encompassing the injury site, they were stabilized with a gelatin‐agarose gel (Jones & Calabresi, 2007). Subsequently, the embedded samples were fixed in 4% paraformaldehyde and then dehydrated in ethanol. Then, the nerves were split along the longitudinal axis.

**FIGURE 1:**
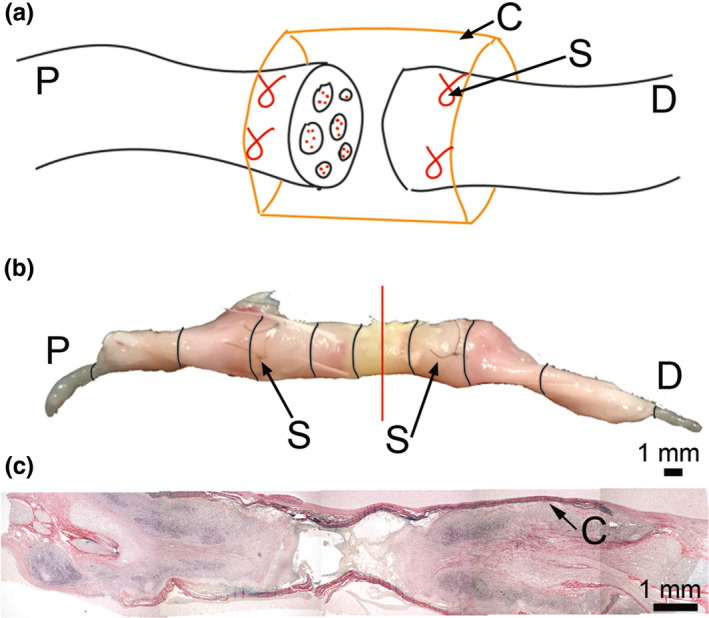
An experimental model of nerve injury. (a) A schematic depicting bridging the proximal (P) stump with the distal (D) stump of a transected sciatic nerve using the Axoguard Nerve Connector conduit (C). Placing the sutures (S) secures the conduit to the margins of the sumps. (b) An isolated nerve fragment encompassing the site of transection indicated by a vertical line. The consecutive sections of the nerve that were analyzed are also indicated. (c) Histology of the longitudinal section encompassing the injury site of the nerve harvested 4 weeks after injury

While one longitudinal segment of the nerve was used to prepare the longitudinal histological sections, the second longitudinal segment was sliced to generate cross‐sections (Figure 1). First, this segment was divided approximately in the middle of the injury site to generate the proximal fragment and the distal fragment. Next, these fragments were separated into ~2‐mm consecutive segments (Figure 1). Finally, all samples were marked and then embedded in the paraffin blocks.

### 2.5 Histological assays

To monitor scar formation and degeneration of the myelin sheath simultaneously, the samples were double‐stained with luxol fast blue (LFB) for myelin and picrosirius red for collagen fibrils. Subsequently, the samples were counterstained with hematoxylin. A microscope (Eclipse E600; Nikon, Inc.) was used to observe and photograph the nerve specimens. In addition, the samples stained only with picrosirius red were analyzed with a polarized light microscope (Eclipse LV100POL; Nikon Inc.), as described (Rivlin et al., 2017; Steplewski et al., 2016).

The specificity of staining of myelin with LFB is well documented, and this staining overlaps with immunostaining of myelin‐specific proteins (Carriel, Garzon, Alaminos, & Campos, 2011; Kluver & Barrera, 1953; Rivlin et al., 2017; Xiang et al., 2005). Similarly, picrosirius red is a collagen‐specific dye with high affinity to the triple‐helical structure of fibrillar collagens (Junqueira, Bignolas, & Brentani, 1979). Thus, employing these staining techniques provided a reliable method to monitor changes in both parameters simultaneously.

### 2.6 Immunohistology

The samples underwent immunohistology to visualize cells associated with the production of the scar tissue in response to nerve injury (Rivlin et al., 2017). In brief, we analyzed the following two proteins: (a) heat shock protein 47 (HSP47), a protein chaperone that controls the intracellular process of collagen triple helix formation and whose expression increases during collagen production, including in fibrosis and neural scarring, and (b) αSMA, a protein naturally present around the blood vessels and in pericytes, perineurial fibroblasts, and myofibroblasts that, in some tissues, contributes to the formation of fibrotic scars (Naitoh et al., 2001; Penke & Peters‐Golden, 2019; Rivlin et al., 2017; Taguchi & Razzaque, 2007). To visualize the axons, we analyzed the nerve samples for the presence of a neurofilament‐specific marker.

To detect HSP47, αSMA, or a pan‐axonal neurofilament marker, we employed the following primary antibodies: (a) mouse monoclonal anti‐HSP47 (Santa Cruz Biotechnology, Inc.), (b) mouse monoclonal anti‐αSMA (Abcam), and (c) mouse monoclonal anti‐neurofilament marker (BioLegend Inc.). Subsequently, the primary antibodies were detected with specific secondary anti‐mouse antibodies conjugated to Alexa Fluor 594 or Alexa Fluor 488 (LifeSciences/Thermo Fisher Scientific).

Sections of the nerves were double‐stained for both HSP47 and αSMA using mouse monoclonal anti‐HSP47 antibody (Santa Cruz Biotechnology, Inc.) and goat polyclonal anti‐αSMA antibody (Invitrogen/Thermo Fisher Scientific). While HSP47 was visualized with a red fluorophore (Alexa Fluor 594; LifeSciences/Thermo Fisher Scientific), αSMA was detected with a green fluorophore (Alexa Fluor 488; LifeSciences/Thermo Fisher Scientific). Negative controls, in which primary antibodies were omitted, were also prepared and analyzed. These controls were prepared to exclude a possibility of positive staining due to nonspecific binding of the secondary antibodies. Following immunostaining, all samples were treated with 4',6‐diamidino‐2‐phenylindole (DAPI) to stain the nuclei. A fluorescence microscope (Eclipse E600; Nikon Inc.) was employed to observe immuno‐stained samples. Table S1 summarizes the immunostaining conditions used to detect the above protein markers.

Green fluorescence filter was utilized to observe infiltrating eosinophils that fluoresce due to the presence of flavin adenine dinucleotide (FAD) and erythrocytes whose green autofluorescence is caused by peroxidation (Khandelwal & Saxena, 2007; Mayeno, Hamann, & Gleich, 1992; Weil & Chused, 1981). In some samples, green autofluorescence also allowed us to detect the extracellular matrix‐rich regions of the nerves. This autofluorescence is mainly due to the cross‐links that stabilize the fibrillar structure of collagen‐rich tissues (Fujimoto, 1977; Monici, 2005).

### 2.7 Quantification of the αSMA‐specific and the HSP47‐specific staining

As indicated above, we focused on the 4‐week‐old rabbits in which we analyzed the relative contents of the αSMA‐positive and the HSP47‐positive cells present in the distinct regions of the healing nerves. We defined the following regions: (i) the intraluminal compartment of the conduit, (ii) the extraluminal compartment of the conduit, (iii) the proximal stump of the transected nerve, (iv) the distal stump of the transected nerve, and (v) the intraneural area of an uninjured nerve (no extraneural scar present; no proximal and distal sites defined).

NIS Elements image analysis software (Nikon Inc.) was used to quantify the αSMA‐positive and the HSP47‐positive cells. In brief, specific areas of the cross‐sections of the nerves (i.e., intraluminal fascicles and extraluminal fibrotic tissue) were first selected by outlining regions of interest (ROI). Subsequently, the areas occupied by cells positive for αSMA or HSP47 were measured. Finally, the percent of each ROI occupied by the αSMA‐positive and the HSP47‐positive cells was calculated for each ROI.

On average, we analyzed 8 histological sections (4 stained for αSMA and 4 stained for HSP47) per one proximal and one distal stump of a 4‐week‐old rabbit. Those 8 sections covered about 5–8 mm of each stump, measuring from the site of the incision. Please note that obtaining nerve sections of identical dimensions was virtually impossible due to the varying geometries of the sites of nerve injury and surrounding scar tissue formed in different rabbits.

The results were evaluated using ANOVA, with *p* ≤ .05 considered statistically significant (univariate ANOVA, IBM SPSS Statistics v. 26; IBM Corp.). We examined the main effects of specific locations on the relative amounts of the αSMA‐positive and the HSP47‐positive cells. Also, we examined the interaction effect of the luminal localization (i.e., intraluminal vs. extraluminal) and the position of a stump (i.e., proximal vs. distal) on the relative content of the αSMA‐positive or the HSP47‐positive cells present in the analyzed regions.

### 2.8 Fourier‐transform infrared spectroscopy

For the fourier‐transform infrared (FTIR) spectroscopy, 5‐μm thick sections of the proximal and distal stumps were prepared from the paraffin‐embedded samples. The sections were placed on MirrIR low‐e microscope slides to allow assays in the reflective mode (Kevley Technologies).

Following deparaffinization, we scanned the samples using an FTIR spectrometer (Spotlight 400; Perkin Elmer), as previously described (Steplewski et al., 2019). The imaging mode was set at 4,000 to 748 cm^−1^ wavenumber spectral range, pixel resolution at 25 µm, with 32 scans per pixel, and spectral resolution at 8 cm^−1^.

Following scanning the samples, we demonstrated the distribution and the intensities of the collagen‐specific signals from the absorbance peak centered around 1,338 cm^−1^ (attributed to the CH_2_ wagging vibration of proline side chains; Camacho, West, Torzilli, & Mendelsohn, 2001; Riaz et al., 2018).

Because the collagen content in the conduits' walls was relatively high in comparison to the content within the nerve tissue, the absorbance scale was set at a range of 0–0.45 units. Thus, sites with collagen content above the value of 0.45 appeared as white areas (see results). Applying these settings, however, allowed us to utilize the full‐range of colors to mark areas of the scar tissue with varying collagen content.

Measuring the area of the absorbance of the amide I peak of the infrared protein spectrum centered around 1,650 cm^−1^ (attributed to the C=O stretching vibration) provides valuable information about the content of proteins within analyzed regions. Analyzing the ratios of the amide I‐specific and collagen‐specific peaks (AI/C ratio) provides a reliable way to analyze a relevant collagen content directly in a tissue sample (Camacho et al., 2001). To calculate these ratios, multiple areas of the nerves were scanned. Then, each scan was used to generate FTIR spectra (Spectrum Image software; PerkinElmer, Inc.). Subsequently, employing the Spectrum software (PerkinElmer, Inc.), we calculated the AI/C ratios of the areas of the integrated amide I peak centered around 1,650 cm^−1^ and of the collagen peak centered around 1,338 cm^−1^, as described (Bi, Yang, Bostrom, & Camacho, 2006; Kim et al., 2010; Steplewski et al., 2019; West, Bostrom, Torzilli, & Camacho, 2004). Please note that the value of the AI/C ratio is inversely proportional to the relative collagen content.

The AI/C ratios of the following areas of the nerves were calculated and analyzed: (a) intraluminal area of the proximal injured stumps, (b) intraluminal area of the distal injured stumps, (c) extraluminal area of the injury site of the proximal stump, (d) extraluminal area of the injury site of the distal stump, and (e) intra‐neural area of an uninjured nerve (no extraneural scar present; no proximal and distal sites defined). The results were evaluated using ANOVA, with ≤0.05 considered statistically significant (univariate ANOVA, IBM SPSS Statistics v. 26).

## 3 RESULTS

### 3.1 Morphological assays of the proximal and the distal sections of the sciatic nerve

**FIGURE 2:**
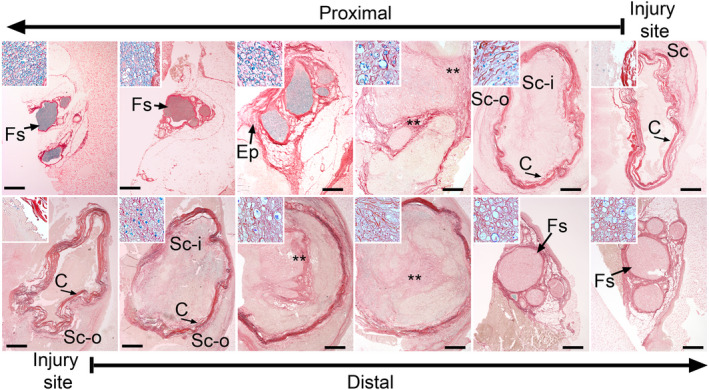
Histology of the proximal (upper row) and the distal (lower row) stumps of the sciatic nerve harvested 4 weeks after injury. The nerves were costained with myelin‐specific and collagen‐specific dyes. The histological sections are derived from the consecutive fragments of the stumps. The arrows at the top and the bottom of the panels indicate the order of the section from the injury site toward the internal regions of the stumps. The inserts show a detailed view of analyzed regions. Symbols: Fs, fascicles; Ep, epineurium; Sc‐i, scar tissue inside conduit; Sc‐o, scar tissue outside conduit; C, conduit; asterisks seen in some panels indicate aggregates of collagen fibrils. Bars = 200 µm

**FIGURE 3:**
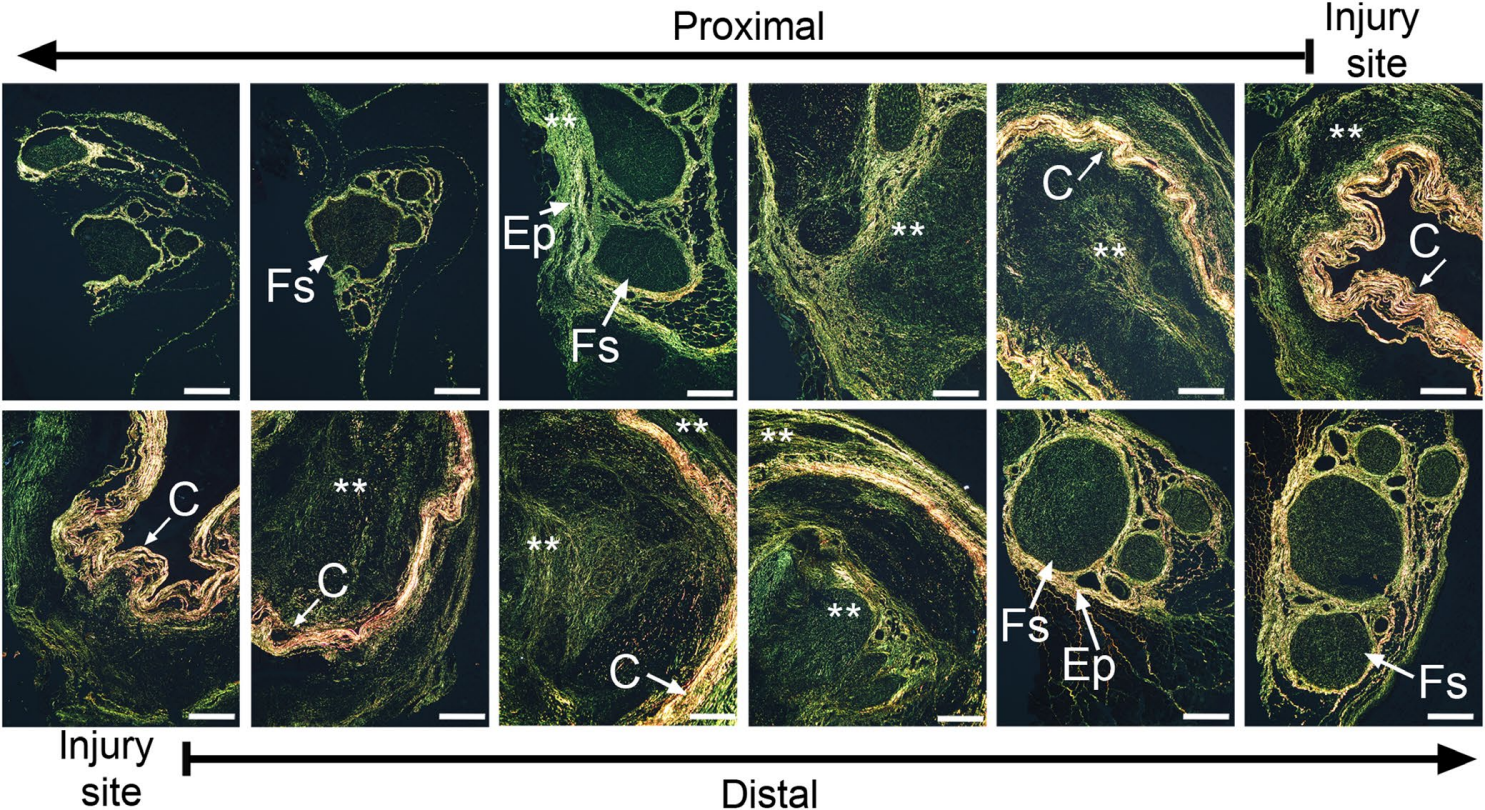
Picrosirius staining of collagen‐rich deposits formed in the proximal (upper row) and the distal (lower row) stumps of the sciatic nerve harvested 4 weeks after injury. Symbols: Fs, fascicles; Ep, epineurium; C, conduit; asterisks seen in some panels indicate aggregates of collagen fibrils. Bars = 200 µm

Collagen‐specific staining of the proximal and the distal sections (Figure 1) collected 4 weeks after surgery demonstrated the formation of fibrotic deposits both inside and outside the conduits (Figures 2 and 3). Consistent with the morphology of the longitudinal sections (Figure 1), at the very middle of the gap region, we did not observe any significant accumulation of the collagen‐rich matrix inside the conduits. In contrast, the fibrotic deposits were present outside the conduits (Figures 2 and 3).

Moving 5–8 mm from the edges of the incisions deeper into the proximal and the distal stumps, we observed accumulation of the fibrotic tissue both outside and inside the conduits (Figures 2 and 3). Consistent with the Wallerian degradation, weak staining of the myelin indicated the degeneration of neurons in the distal stump (Figure 2). In contrast, the myelinated neurons were readily visible in the proximal stump (Figure 2).

At 6 and 10 weeks after injury, analysis of the injury region demonstrated fibrillar deposits, abundantly present both inside and outside the conduit, as well as the original gap region (data not shown). We did not observe any significant presence of cells within the walls of the conduits at any time point of this study.

### 3.2 Organization of the fibrotic tissue

**FIGURE 4:**
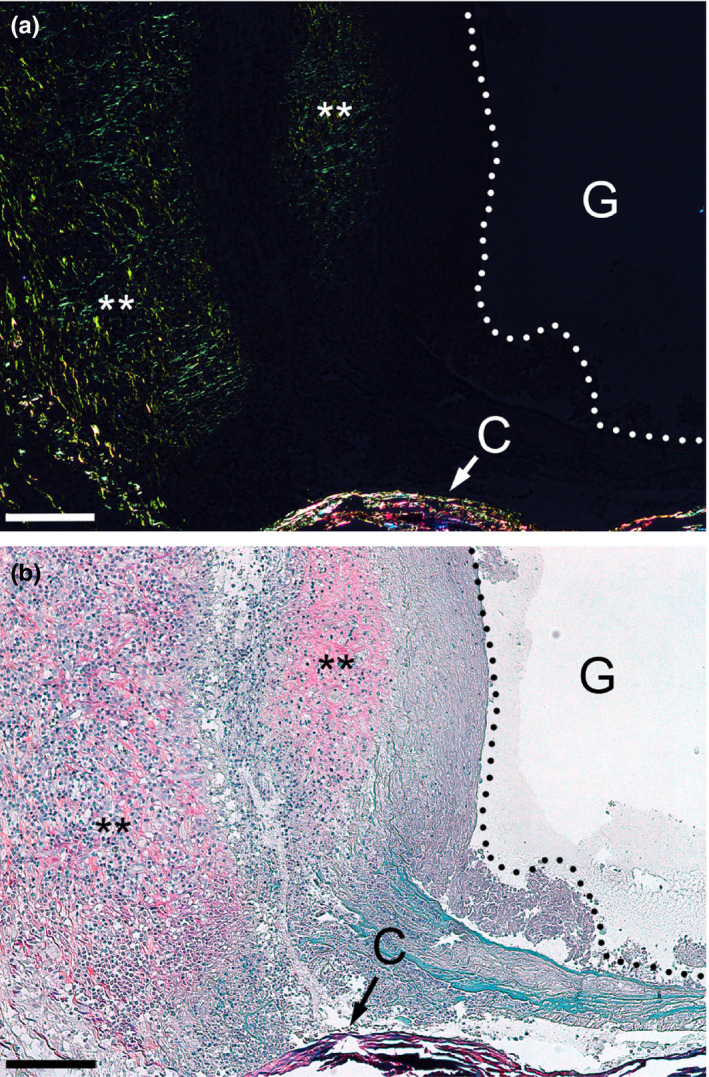
The longitudinal view of the margin of a stump from a transected nerve observed 4 weeks after injury. (a) Picrosirius staining of collagen fibrils formed in the healing sciatic nerve. (b) A view of the region in natural light the cellularity in the context of fibrillar deposits. Asterisks identify corresponding regions in (a) and (b). The dotted line marks the edge of the stump. Symbols: C, conduit; G, the gap between the stumps. Bars = 100 µm

Morphological assays of samples collected 4 weeks after injury demonstrated the deposition of collagen‐rich fibrotic tissue within and around the nerves (Figures 2 and 3). Although at that particular time point, the gap was not fully closed, the fibrotic tissue formed within the stumps (Figures 1, 2, 3 and 4). In the longitudinal sections, the leading edges of the collagen‐rich deposits appeared to form some distance from the very edge of a stump (Figure 4).

Two distinct patterns of organization of collagen fibrils were readily apparent: (a) circumferentially organized fibrils accumulated outside the conduits around the conduit, and (b) transversely organized fibrils accumulated inside the cross‐sectioned conduits (Figure 3). Similar patterns of organization of the fibrils were seen in both the proximal and the distal stumps.

### 3.3 FTIR assays of collagen‐rich deposits

**FIGURE 5:**
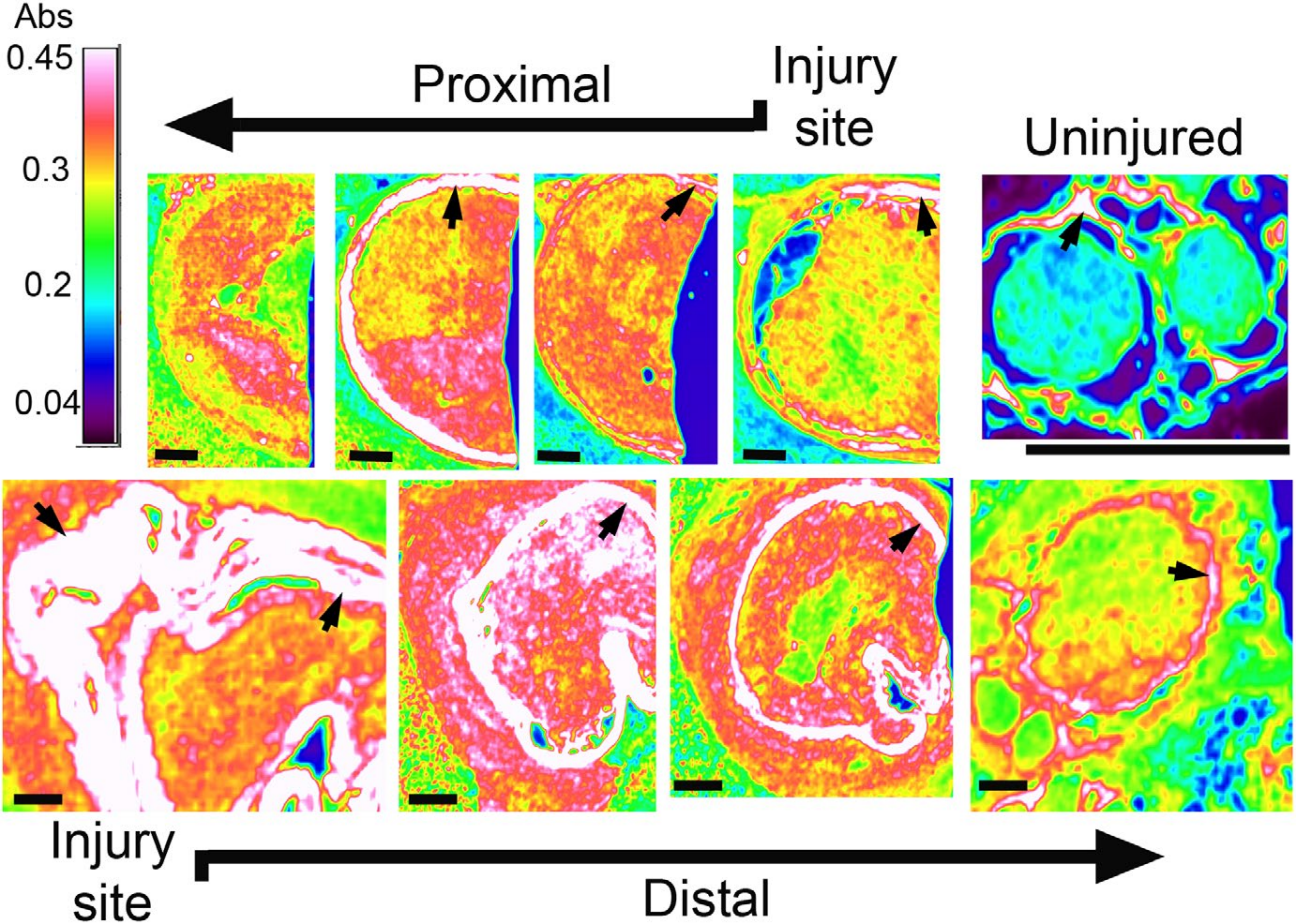
Fourier‐transform infrared spectroscopy assays of the cross‐sections of injured nerves collected for weeks after surgery. The images depict the intensity of the collagen‐specific signal at 1,338 cm^−1^. The absorbance scale was set from 0 to 0.45 units so that the range of colors within the scale represented varying signal intensities. Note that signals stronger than 0.45 units, for example, collagen‐rich conduits (arrows) and fragments of epineurium, appear white. Upper panels depict consecutive regions of a proximal stump and the lower panels show consecutive regions of a distal stump. An uninjured nerve is also presented. Bars = 500 µm

While the picrosirius staining revealed the organization and distribution of the collagen fibrils, FTIR spectroscopy provided more quantitative information on the collagen‐rich deposits (Figure 5). We compared the relative amounts of collagen deposits, expressed as the AI/C ratios, in the proximal and distal segments encompassing the injury site. These segments covered about 5–8 mm of each stump when measured from the edges of the stumps. Within the proximal and the distal segments, we compared the relative collagen content measured within the intraluminal and the extraluminal compartments.

The main effect of the stump location (i.e., proximal vs. distal) was statistically significant where the relative collagen content was higher in the proximal stump than in the distal stump (*F*(1,34) = 20, *p* < .0001). The main effect of the luminal compartment (i.e., intraluminal vs. extraluminal) was also statistically significant, where the relative collagen content in the extraluminal space was lower than in the intraluminal space (*F*(1,34) = 4.28, *p* = .046). There was also a statistically significant interaction between the stump location (i.e., proximal and distal) and the type of a luminal compartment (i.e., intraluminal and extraluminal) on the relative collagen content (*F*(1,34) = 12.335, *p* = .001).

As control uninjured nerves include neither the extraneural scar tissue nor the proximal or distal segments defined by the incision site, we compared the relative collagen content in the intraluminal space of the injured nerves with that of the intrafascicular space in control nerves. We found that the collagen content was higher in the regions encompassing the sites of injury than in healthy control (*F*(1,34) = 38.113, *p* < .0001). Table S2 demonstrates the means and standard deviations of the AI/C ratios used to measure the relative collagen content in analyzed samples.

### 3.4 Patterns of distribution of cells expressing αSMA and HSP47 markers

**FIGURE 6:**
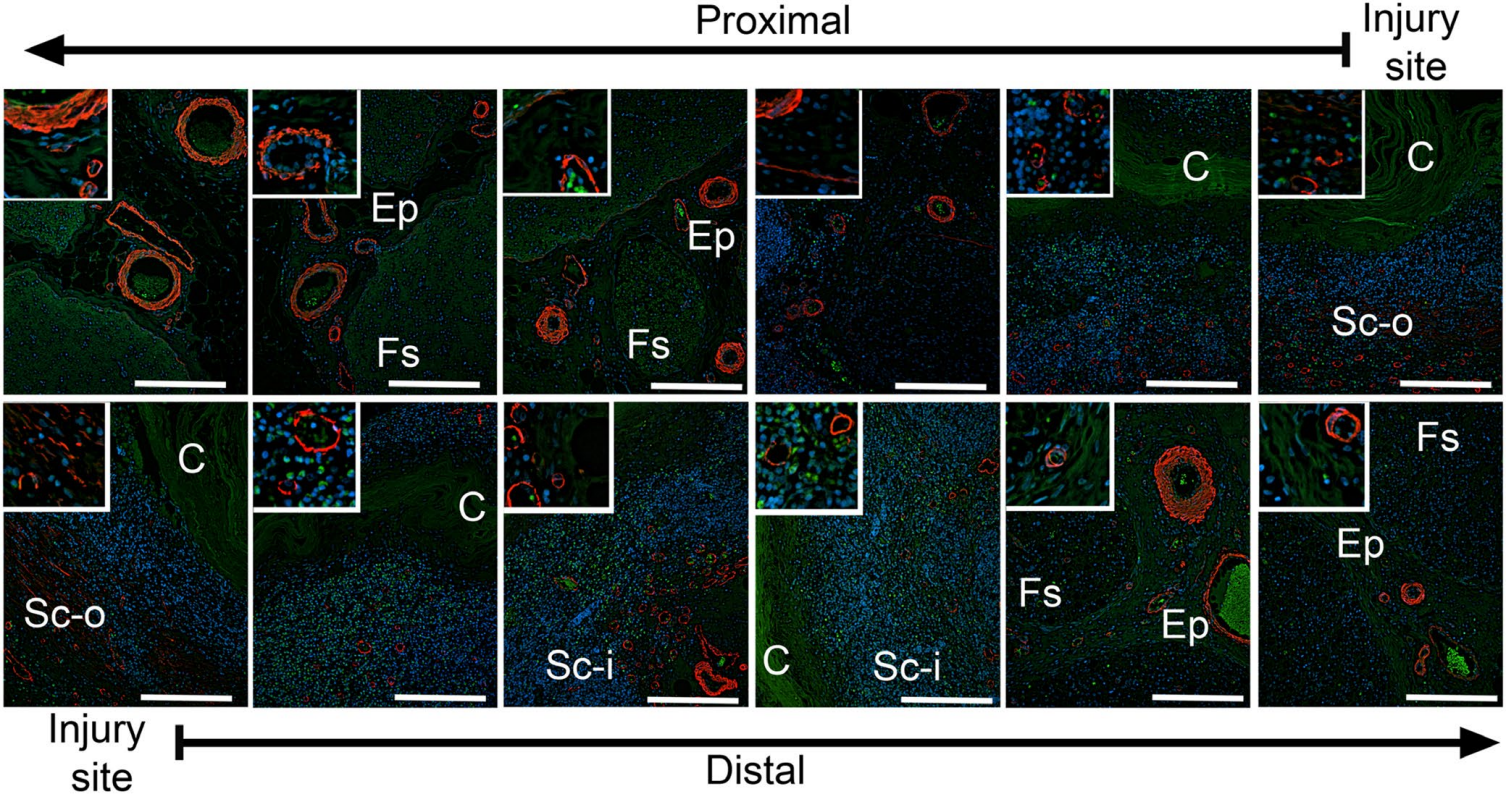
Immunostaining of the proximal (upper row) and the distal (lower row) stumps of the sciatic nerve harvested 4 weeks after injury to detect αSMA. The nerves were costained with 4',6‐diamidino‐2‐phenylindole (DAPI). The histological sections are derived from the consecutive fragments of the stumps. The panels show the localization of αSMA limited to the blood vessels. The arrows at the top and the bottom of the panels indicate the order of the section from the injury site toward the internal regions of the stumps. The inserts show a detailed view of the αSMA‐positive cells. Symbols: Fs, fascicles; Ep, epineurium; Sc‐i, scar tissue inside conduit; Sc‐o, scar tissue outside conduit; C, conduit; asterisks seen in some panels indicate aggregates of collagen fibrils. Bars = 200 µm

As invading fibroblasts produce collagen‐rich scar tissue at the injury site, we studied the distribution of markers that define the collagen‐producing cells. Cross‐sectional analysis of the nerves collected 4 weeks after injury revealed the presence of the αSMA‐positive signals outside and inside the conduits. In both locations, the αSMA‐positive signals were observed around the blood vessels (Figure 6). At 6 and 10 weeks after injury, the cells expressing αSMA exhibited a similar distribution pattern (data not shown). Furthermore, the αSMA‐positive staining was visible in the perineurial fibroblasts (data not shown) in which this marker is a natural intracellular component (Rivlin et al., 2017).

Depending on the orientation of the blood vessels, the αSMA‐positive signals appeared aligned with either the vessel's circumference (vessels oriented perpendicularly to the plane of the cross‐section) or its length (a vessel aligned parallel to the plane of cross‐section). Both the perpendicular and the parallel orientations of the vessels were confirmed by observing the longitudinal sections (Figure S1). We observed that 4 weeks after surgery, the blood vessels did not reach the very edges of the stumps (Figure S1). In contrast, HSP47‐positive cells were present through the entire corresponding region of the nerve (Figure S2).

### 3.5 Quantification of the αSMA‐positive and the HSP47‐positive cells in defined regions of the injured nerves

We also performed quantitative assays of the amount of the αSMA‐positive cells as the function of their location in the proximal and the distal stumps, as well as within the intraluminal and the extraluminal spaces of the conduits. The main effect of the stump location (i.e., proximal vs. distal) on the area occupied by the αSMA‐positive cells was statistically significant such that the proximal stump contained a higher percentage of the αSMA‐positive cells than the distal stump (*F*(1,58) = 4.746, *p* = .033). The main effect of the luminal location (i.e., internal vs. external) on the percent area occupied by the αSMA‐positive cells was not statistically significant (*F*(1,58) = 0.114, *p* = .737). The percent area occupied by the αSMA‐positive cells present in the extraluminal space, however, trended higher compared with the intraluminal space (Table S2) There was no a statistically significant interaction between the stump location and the localization of the luminal compartments (*F*(1,58) = 0.311, *p* = .597).

**FIGURE 7:**
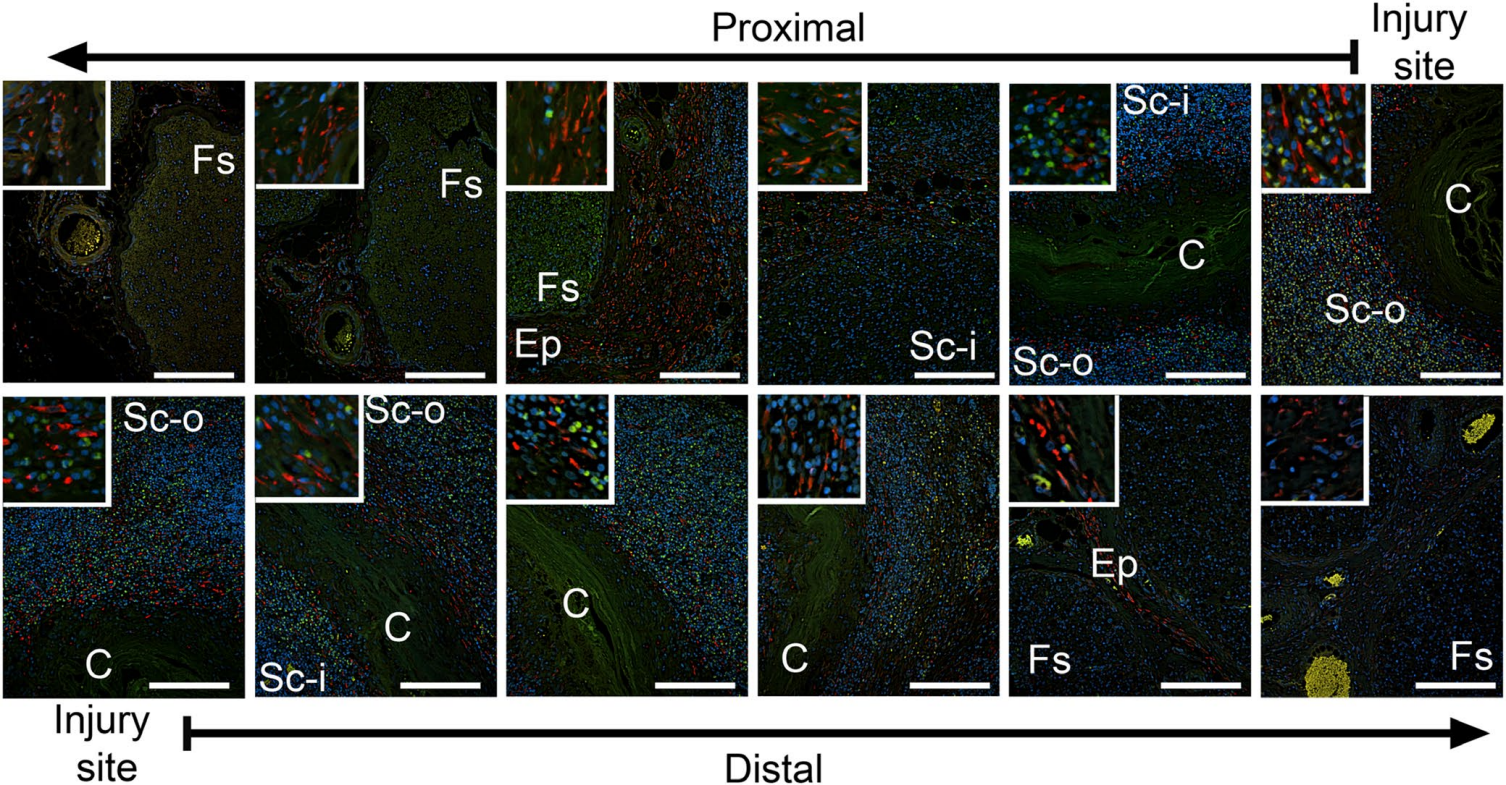
Immunostaining of the proximal (upper row) and the distal (lower row) stumps of the sciatic nerve harvested 4 weeks after injury to detect HSP47. The nerves were costained with DAPI. The histological sections are derived from the consecutive fragments of the stumps. The panels show HSP47 signals in fibroblasts located primarily within the interfascicular epineurium and the epifascicular epineurium. The arrows at the top and the bottom of the panels indicate the order of the section from the injury site toward the internal regions of the stumps. The inserts show a detailed view of the HSP47‐positive cells. Symbols: Fs, fascicles; Ep, epineurium; Sc‐i, scar tissue inside conduit; Sc‐o, scar tissue outside conduit; C, conduit; asterisks seen in some panels indicate aggregates of collagen fibrils. Bars = 200 µm

Since control nerves included neither the extraneural scar tissue nor the proximal or the distal segments defined by the incision site, we compared the percentage of the αSMA‐positive cells present in the intraluminal space of the injured nerves with that of the intrafascicular space in control nerves. The relative content of the αSMA‐positive cells in analyzed ROIs in the regions encompassing the sites of injury was significantly higher than in healthy control (*F*(1,53) = 5.886, *p* = .019). In contrast to the vessel‐associated distribution of the αSMA‐positive signals, the HSP47‐positive fibroblasts were widely distributed in the scar tissue formed outside and inside the conduits (Figure 7; Figure S2). Table S2 demonstrates the means and standard deviations of analyzed parameters.

Quantitative assays were also performed to measure the percentage of the HSP47‐positive cells in the proximal and the distal stumps, as well as within the intraluminal and the extraluminal spaces of the conduits. The percentage of the ROI area occupied by the HSP47‐positive cells was significantly higher in the proximal stump than in the distal stump (*F*(1,41) = 27.1, *p* < .0001). Similarly, the percentage of the HSP47‐positive cells was significant higher in the extraluminal space than in the intraluminal space (*F*(1,41) = 15.452, *p* < .0001). There was no statistically significant interaction between the stump location and the luminal compartment (*F*(1,41) = 0.072, *p* = .79).

We also compared the percentage of the HSP47‐positive cells present in the intraluminal space of the injured nerves with that of the intrafascicular space in control nerves. The relative content of HSP47‐positive cells was significantly higher in the analyzed ROIs in the regions encompassing the sites of injury than in the healthy control (*F*(1,37) = 22.934, *p* < .0001). Finally, in all of the analyzed regions encompassing the sites of injury, the overall percentage of the ROIs occupied by the HSP47‐positive cells was higher than the percentage occupied by the αSMA‐positive cells (*F*(1,91) = 124.9, *p* < .0001). Table S2 presents the means and standard deviations of analyzed parameters.

### 3.6 Distribution of myofibroblasts

**FIGURE 8:**
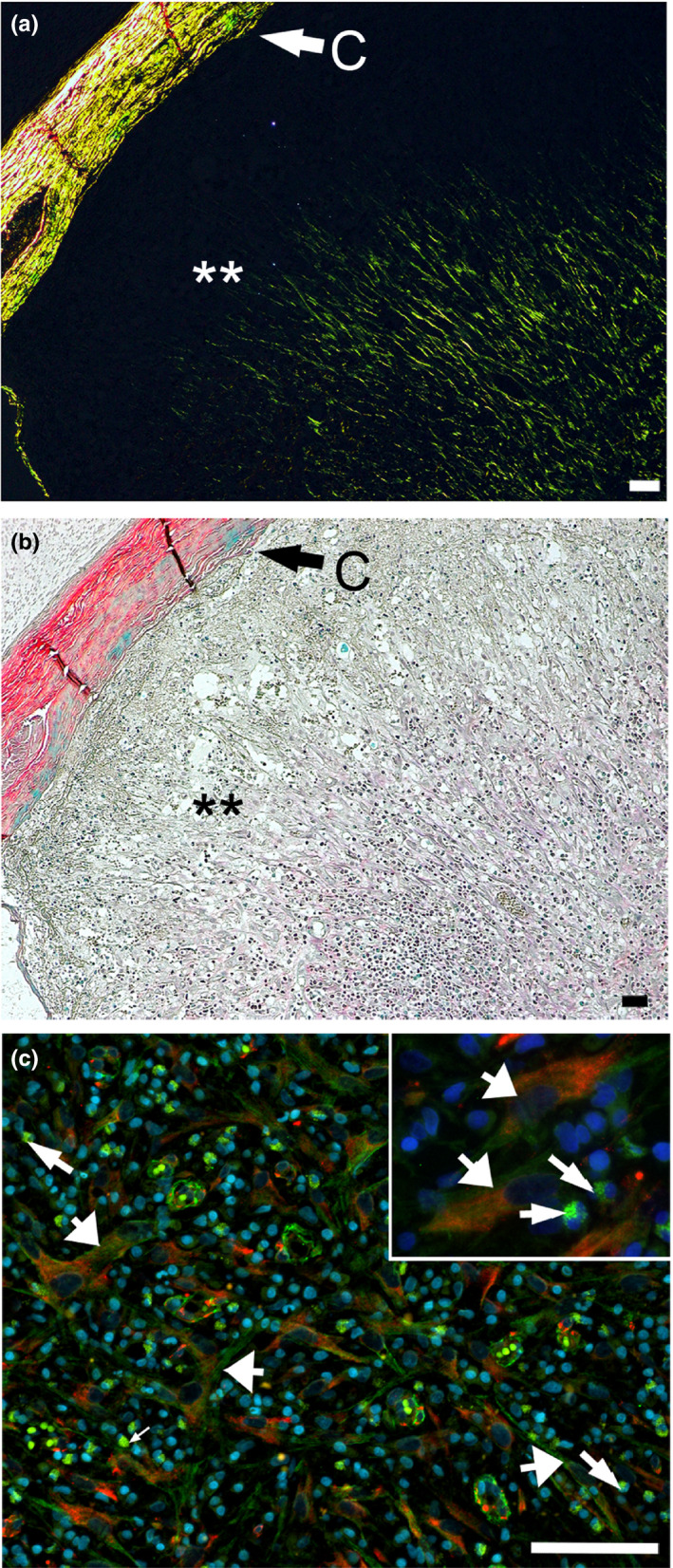
Double‐immunostaining of the cross‐sections of a nerve collected 4 weeks after injury for the presence of the αSMA (green)/HSP47 (red)‐positive myofibroblasts. The cross‐sections are derived from the margin region of a stump. (a) Picrosirius red staining of collagen fibrils seen in polarized light inside the conduit (C). (b) The same region observed in nonpolarized light to visualize the cellularity. (c) Double staining depicting the αSMA/HSP47‐positive myofibroblasts; the insert presents double‐positive myofibroblasts (wide arrows) in the context of inflammatory cells (narrow arrows). Asterisks mark the area depicted in (c). Bars = 50 µm

**FIGURE 9:**
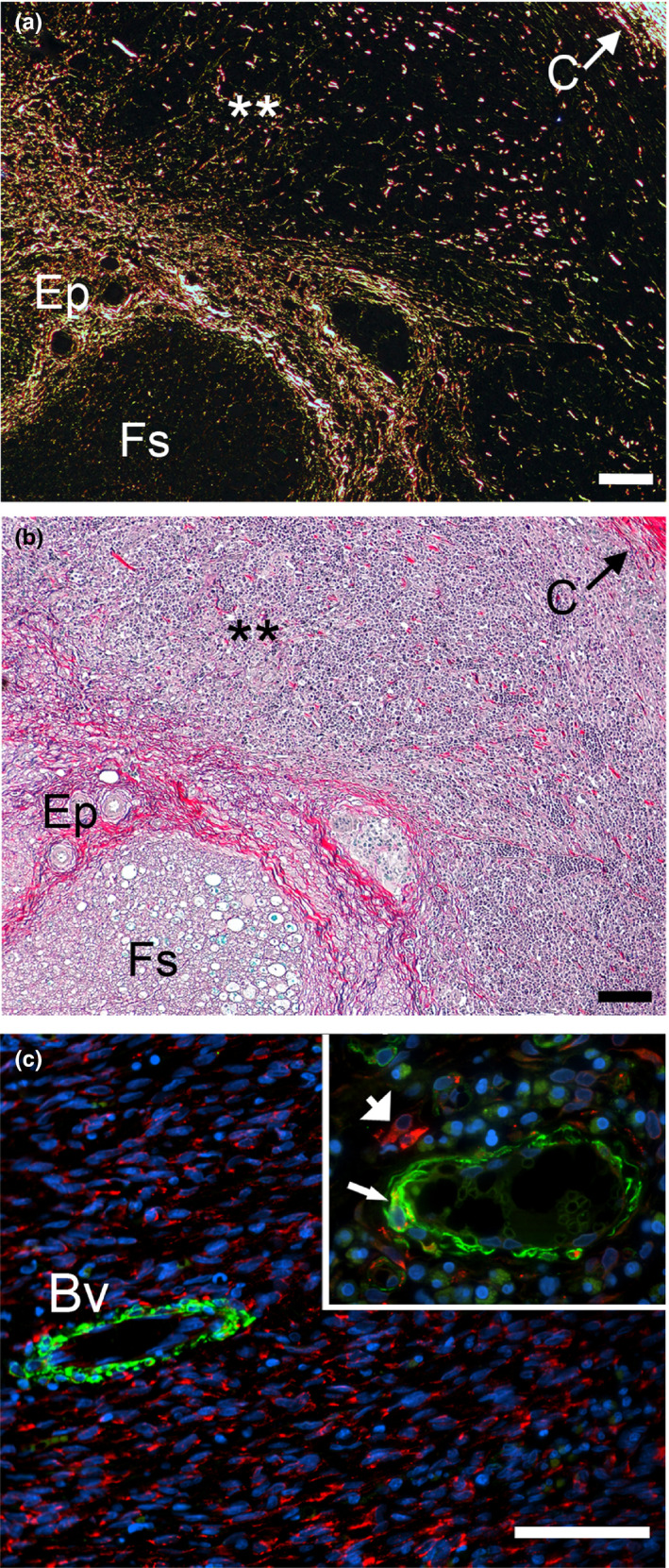
Double‐immunostaining of the cross‐sections of a nerve collected for weeks after injury for the presence of the αSMA (green)/HSP47 (red)‐positive myofibroblasts. In comparison to the margin area depicted in Figure 4, the cross‐section presented here is located in the dipper region of a stump. (a) Picrosirius red staining of collagen fibrils seen in polarized light inside the conduit (C). (b) The same region observed in nonpolarized light to visualize the cellularity. (c) Double staining depicting the αSMA‐positive/HSP47‐negative and the αSMA‐negative/HSP47‐positive cells; the insert presents the HSP47‐positive fibroblasts (wide arrows) and the αSMA‐positive cells seen in the blood vessels (narrow arrows). Asterisks mark the area depicted in (c). Symbols: C, conduit; Ep, epineurium; Fs, fascicle; Bv, blood vessel. Bars = 100 µm

Because tissue‐embedded αSMA‐positive myofibroblasts that are not a part of the blood vessels participate in wound healing and fibrosis of many tissues and organs, we analyzed the nerve samples for the presence of those cells. Immunostaining of the nerve samples demonstrated the presence of the αSMA/HSP47 double‐positive cells only in the areas of the margins of the stumps (Figure 8). Careful observation of deeper regions of the stumps did not reveal any double‐positive cells. Instead, double staining confirmed two separate groups of cells present in these regions: (a) the αSMA‐positive/HSP47‐negative cells that were associated only with the blood vessels, and (b) the HSP47‐positive/αSMA‐negative fibroblastic cells that densely populated the stumps (Figure 9).

We observed that the αSMA‐positive/HSP47‐positive myofibroblasts appeared in the cap areas with sparse collagen fibrils (Figure 8). In contrast, in the deeper regions of the stumps where the αSMA‐positive/HSP47 double‐positive myofibroblasts were virtually absent, the fibrillar deposits were quite dense, as demonstrated by polarized light microscopy of the picrosirius red‐stain samples (Figure 9).

### 3.7 Formation of the scar tissue around the sutures

**FIGURE 10:**
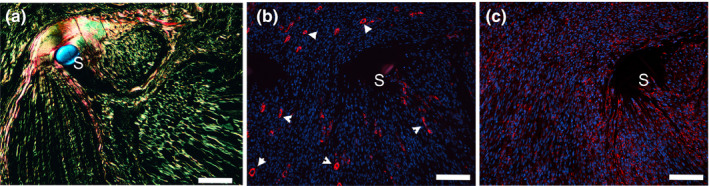
Immunostaining for the presence of αSMA‐positive and HSP47‐positive cells in the scar tissue formed around sutures (S) used to secure conduits to the stumps. (a) A polarized light image of the picrosirius red‐stained collagen‐rich scar tissue. (b) Staining for the presence of αSMA‐positive cells (arrows). (c) Staining for the presence of HSP47‐positive cells. Bars = 50 µm

We also analyzed the αSMA‐positive and the HSP47‐positive signals in the areas surrounding the sutures used to secure the conduits to the nerve stumps. As indicated in Figure 10, there was a substantial accumulation of collagen‐rich fibrotic deposits around the suture that appeared to be under tension. While on one side of the suture, the fibrotic tissue formed a thick acellular band; on the opposite side of the suture, the collagen fibers were aligned along clear tension lines. Of note, despite the apparent tension imposed by the suture, no αSMA‐positive fibroblasts were observed in the suture area. Similar to other regions of the stumps, αSMA‐positive staining was only observed in the blood vessels. The regions around the sutures, however, were densely populated by fibroblasts that actively produced collagen, as evident by the HSP47‐positive staining (Figure 10).

### 3.8 Growth of neural filaments

**FIGURE 11:**
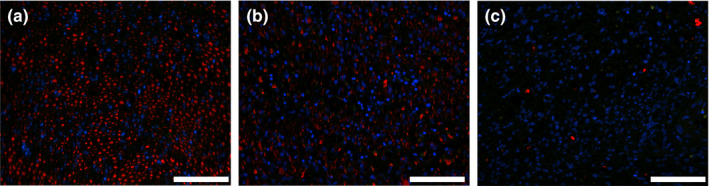
Immunostaining of neurofilaments in an uninjured sciatic nerve (a), as well as the proximal (b) and the distal (c) stumps of the injured nerve, collected 4 weeks after transection. Bars = 100 µm

Analysis of neural tissue collected 4 weeks after surgery demonstrated that neural filaments were present in the proximal segment and virtually absent in the distal segments, thus consistent with the Wallerian degenerative process. The presence of the filaments in the uninjured nerve confirmed the specificity of immunostaining (Figure 11). Observation of the distal stumps isolated after 6 and 10 weeks after surgery indicated neural filaments were present, thereby indicating regeneration of the sciatic nerve (Figure S3).

Morphological data and results presented here apply to all analyzed animals. To avoid redundancy and stay within limits of a scientific publication, we only present representative results and do not include all morphological data from all animals employed in this study.

### 3.9 Summary of results

**FIGURE 12:**
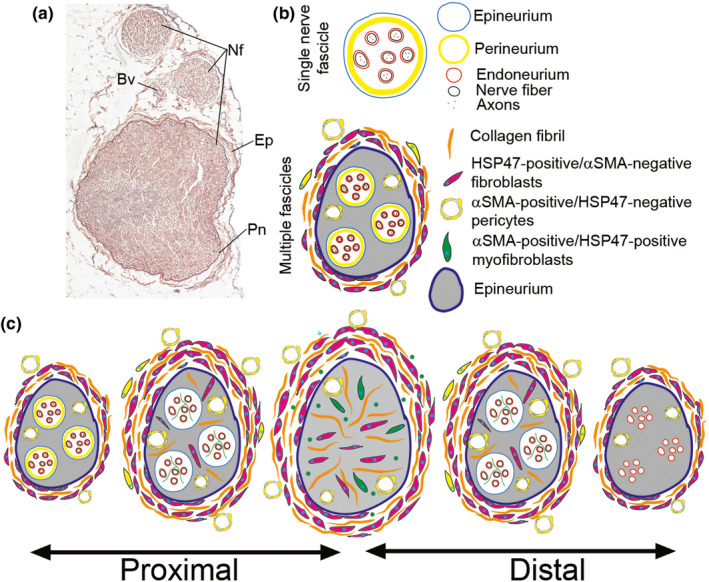
A schematic summary of the scar region of the sciatic nerve for weeks after injury. The schematic depicts crucial elements of the scar, including blood vessels, collagen fibrils, and cells that participate in the scar formation process

Figure 12 summarizes our results in the context of analyzed regions of the nerves.

## 4 DISCUSSION

The overall goal of this study was to broaden our understanding of the patterns of formation of neural scars after transection injury and surgical repair of a model sciatic nerve. We considered the presence of a conduit often used by clinicians to repair injured nerves. As the presence of a conduit compartmentalizes the site of injury into the intraluminal and the extraluminal spaces, we studied the formation of the scar tissue within these distinct domains. Furthermore, we analyzed the distribution patterns of cells that contribute to the production of the collagen‐rich scar tissue. Overall, the results of our studies indicate that compartments created by the presence of the conduits differed in the number of collagen‐producing cells and the relative amounts of collagen‐rich scar deposits. We determined that the proximal site of the injured nerves showed the most active scarring activity within the experimental timeframe selected in this study.

Like the scars formed in other tissues, those that develop due to injury of the peripheral nerves mainly comprise collagen‐rich fibrils (Salonen et al., 1985). Although collagen fibrillogenesis is a crucial element of nerve regeneration, the excessive deposition of the collagen‐rich matrix within and around the injured peripheral nerve represents a significant clinical problem (Atkins et al., 2006; Koopmans, Hasse, & Sinis, 2009). In particular, neural scars formed in response to injury impair nerve gliding and block axonal growth.

In physiological conditions, Schwann cells and fibroblasts that dwell in peripheral nerves produce fibril‐forming and network‐forming collagens that build the mechanical scaffold of the nerve tissue and provide support for the axons (Koopmans et al., 2009). Our earlier studies on scar formation in crushed and partially transected sciatic nerves indicated that the Schwann cells and epineurial fibroblasts actively produce collagen molecules that form the bulk of the intraneural and the extraneural scars (Rivlin et al., 2017). The exact role and spatial distribution of cells that produce collagen‐rich deposits formed in response to nerve injury, however, is unclear (Chernousov & Carey, 2000).

The αSMA‐positive fibroblastic cells, referred to as myofibroblasts, play a crucial role in the healing of many tissues, including skin, joint capsule, and ocular tissues. While the contractile behavior of these cells helps to close the wounds, they may also accelerate the formation of fibrotic scars by overexpressing elements of the scar tissue, most notably fibrillar collagens (Darby, Zakuan, Billet, & Desmouliere, 2016; Hildebrand, Zhang, & Hart, 2007; Weng et al., 2016; Zhang, Rekhter, Gordon, & Phan, 1994).

In the context of peripheral nerve injury, researchers observed that the αSMA‐positive fibroblasts around the perimeter of the nerve trunk. They suggested that the circumferential orientation of these cells combined with their contractile behavior generates forces constricting the nerve trunk, thereby altering axon regeneration (Chamberlain et al., 2000). Although we observed the presence of some αSMA‐positive myofibroblasts in our earlier study of a model of a partially transected nerve, here, these fibroblasts were mostly absent in the scar tissue. Instead, the presence of αSMA was limited to the blood vessels and perineurium, where this marker is a natural component of pericytes and perineurial fibroblasts, respectively (Rivlin et al., 2017).

Although αSMA‐positive myofibroblasts were not present in the stumps, we cannot exclude the possibility that they were present only transiently outside of our 2‐ to 10‐week postinjury experimental time window. This possibility is in agreement with our study on the crush injury and partial‐transection injury modes, where we observed some αSMA‐positive fibroblasts 1–2 weeks after injury and with Chamberlain et al. (2000), who observed αSMA‐positive myofibroblasts only 60 days after injury (Rivlin et al., 2017).

Moreover, we cannot exclude the possibility that the presence of collagen‐rich conduits employed here to bridge the gap between the proximal and the distal stumps may have inhibited the formation of myofibroblast‐based contractile capsules. Earlier research has supported this notion by showing that, in contrast to conduits fabricated from silicone, collagen‐based conduits limit the number of myofibroblasts in injured nerves (Chamberlain et al., 2000; Darby et al., 2016).

While Spilker, Asano, Yannas, and Spector (2001) suggested that the αSMA‐positive myofibroblasts have contractile functions during peripheral nerve repair, their contribution to the formation of collagen‐rich neural scars is not clear. Our study here suggests that these cells may support the nerve repair process by contracting the wounds rather than producing collagen‐rich matrix. Our observations that αSMA‐positive fibroblasts are only present close to the ends of the stumps where collagen fibrils are sparse supports this suggestion.

The lack of αSMA‐positive fibroblasts within regenerating nerves was a widespread phenomenon. Not only were these cells absent within the nerve tissues, but also, they were absent around the sutures that appeared to cause the mechanical tension. The absence of the αSMA‐positive fibroblasts in those areas is somewhat inconsistent with studies suggesting that mechanical tension stimulates the transdifferentiation of fibroblasts to myofibroblasts (Junker, Kratz, Tollback, & Kratz, 2008). It is likely, however, that in our model system, the tension generated by the sutures did not reach the magnitude needed to induce this kind of transdifferentiation. In studies on fragments of human burn scars, researchers needed 0.66 N/cm^2^ of force to transdifferentiate fibroblasts to the αSMA‐positive fibroblasts (Junker et al., 2008). Furthermore, the matrix stiffness of the neural scar was likely inadequate to induce the differentiation into the αSMA‐positive myofibroblasts. As demonstrated by experiments with gel matrices of different stiffness, fibroblasts readily differentiate to myofibroblasts when cultured in soft matrices (Young's moduli 0.5–1.0 kPa) but not stiff matrices (Young's moduli 10–50 kPa; Huang et al., 2012; Shi et al., 2013).

The low content of αSMA in the neural scars studied here is somewhat surprising considering that αSMA often serves as a marker of active scarring and considering that some studies consider it a significant source of the collagen and other molecules that form scar tissue (Hinz, 2016). Thus, we suggest that αSMA is not a consistent marker of collagen‐producing cells associated with neural fibrosis. This postulation agrees with the study by Sun, Chang, Reed, & Sheppard (2016), who demonstrated that αSMA‐positive fibroblasts in some fibrotic organs, including lung and kidney, represented only a minor fraction of collagen‐producing cells.

In contrast, our studies of the HSP47‐positive fibroblasts revealed that they were abundant in all collagen‐rich areas present outside and inside the conduits. Although our study does not identify the origin of these cells, most of those seen inside the conduits occupied the epifascicular and interfascicular epineurium. This observation aligns with earlier studies suggesting that fibroblasts from the neural connective tissues participate in the healing of injured peripheral nerves (Spilker et al., 2001).

We observed the buildup of the intraneural scar tissue from within the stumps, as determined by the absence of collagen fibrils and reduced cellularity at the very edges of the stumps 4 weeks after injury. This observation may indicate that resident neural fibroblasts are responsible for the production of collagen‐rich deposits within the conduits' lumina. Because we did not observe any cells traversing the walls of the conduits employed here, we also suggest that the external scars produced around the injured nerves, outside the conduits, are a product of fibroblasts that originated from surrounding tissues rather than from the injured nerves themselves.

While quantitative immunohistology provides detailed insight to the distribution of the αSMA‐positive and the HSP47‐positive cells as the function of their spatial localization, FTIR‐based measurements shed light on the relative content of collagen. Overall, histological assays indicate that 4 weeks after injury, the content of the αSMA‐positive and the HSP47‐positive cells was higher in the proximal stumps than in the distal stumps. Similarly, the content of both cell types was higher in the extraluminal space than in the intraluminal space.

Although, when compared to the uninjured nerves, the content of both cell types increased in the injured sites, the increase of the HSP47‐positive cells was more prominent than the increase in αSMA‐positive/HSP47‐negative cells. The increase in the number of cells expressing the HSP47 marker correlated with the increase of the relative collagen content in the injury sites. As indicated by the FTIR‐based assays, this relative content was higher in the proximal site than in the distal site in both the extraluminal and the intraluminal compartments.

More significant scarring activity in the proximal segment, indicated by a higher number of collagen‐producing cells and markedly increased collagen accumulation in comparison with the distal site, represents a novel observation. Future research is warranted to explain whether higher pro‐fibrotic activities in the proximal stump result from retrograde neuronal signals from the distal stump or from local trophic signals from the proximal stump itself. As suggested by Zochodne and Cheng local endoneurial cells of the proximal nerve can be activated by blood‐borne factors due to the local breakdown of the blood‐nerve barrier. They detected several factors with pro‐fibrotic activities, including basic fibroblast growth factor (bFGF), neural growth factor (NGF), and insulin growth factor 1 (IGF‐1; Zochodne & Cheng, 2000). We cannot exclude the possibility that the production of these factors in the proximal stumps could enhance pro‐fibrotic activities not only in the intraluminal space but also, due to diffusion, in the extraluminal compartment.

The scarring process we studied here had direct implications for axonal growth. The rate at which the axons elongate is determined by factors that are intrinsic and extrinsic to the growing axon. While the intrinsic factors include the production of materials needed for nerve regeneration, the extrinsic ones include the physical environment of the regenerating axons, including the presence of the scar tissue (Black & Lasek, 1979). Studies demonstrated that the rabbit axons could grow about 4 mm/day following crush or transection injury (Gutmann, Guttmann, Medawar, & Young, 1942). Researchers pointed, however, that this rate describes the growth within distal stumps and that the axonal growth within the injury site is markedly slower (Black & Lasek, 1979). Considering that at 4 weeks after injury, we did not observe any significant presence of regenerating axons in the distal stumps, we propose that the presence of the scar tissue and the lack of tissue continuity in the gap region could have contributed to weak axon growth. The axons were readily visible in the distal stumps only 6 weeks after injury; at this time, the collagen‐rich tissue filled the gap region.

The observation that the axons traversed quite dense fibrotic tissue and grew into the distal stumps may indicate that the intraneural scars do not form an impenetrable barrier for the axonal growth. Consequently, it may be the extraneural scars that impede the repair of the nerves more significantly than the intraneural scars due to the formation of adhesions, prevention of the gliding of injured nerves, and contraction of the regenerating nerves.

Our research not only reveals the compartmentalization of the scar formation due to the presence of the conduits, but also describes patterns of production of the collagen‐rich deposits within specific compartments. Furthermore, our study defines the role of myofibroblasts in the repair of transected peripheral nerves. These findings provide valuable information about both spatial and cellular targets of anti‐fibrotic approaches aiming at limiting excessive posttraumatic neural scaring.

This study has limitations that we intend to address in future studies: (a) Additional groups of animals must be employed to evaluate the formation of collagen‐rich deposits at earlier and later stages of peripheral nerve regeneration. (b) We have to include assays of noncollagenous elements of the scar tissue to fully understand the processes of nerve repair and the formation of complex neural scars. (c) It will be necessary to introduce a control group in which injured nerves will be repaired without conduits and with conduits fabricated from synthetic materials. One possible option could be to bridge a gap with an autograft or silicone tubes. Introducing these additional groups would make it possible to determine further the role of the conduits in repairing the peripheral nerves. (d) This study does not identify the origin of HSP47‐positive cells. (e) To fully comprehend the role of the scar formation, it will be necessary to perform functional studies of regenerated nerves and compare results as the function of surgical techniques used to bridge the gaps. (f) Although this study was carried out in a relevant animal model, its finding may not fully represent processes occurring in human patients.

Based on our results, it appears that the proximal region of the peripheral nerve at the injury site plays an important role in the scarring processes associated with the formation of collagen‐rich deposits. Scarring after peripheral nerve injury and surgery remains a common clinical problem with substantial morbidity and poor outcomes. It is hoped that a better understanding of the mechanisms that caused peripheral nerve fibrosis will lead to novel treatments that may improve outcomes of treatment in the clinical setting.

## 5 CONCLUSIONS

The results of our study reveal patterns of deposition and the architecture of collagen‐rich scar tissue formed in response to injury of the sciatic nerve. These results indicate significant differences in the scar deposits formed in the proximal and the distal stumps, inside and outside the injured nerves surgically repaired using conduits. Furthermore, our study demonstrated that the role of canonical pro‐fibrotic myofibroblasts in scarring of peripheral nerves might be limited to contracting the wound rather than excessive production of collagen‐rich fibrotic matrices. Further studies will reveal the role and origin of fibroblastic cells responsible for robust scar formation due to the injury of peripheral nerves.

## CONFLICT OF INTEREST

The authors declare no conflict of interest.

## AUTHOR CONTRIBUTION

A. Fertala, J. Fertala, A. Steplewski: Substantial contribution to the acquisition, analysis, and interpretation of data. Contributed to drafting the manuscript. M. Wang, M. Rivlin, P. Beredjiklian: Significant contribution to designing an animal model of sciatic nerve injury and surgical procedures. Substantial contribution to the acquisition and interpretation of data form an animal model. Participated in drafting the manuscript. All authors have read and approved the final submitted manuscript.

### Peer Review

The peer review history for this article is available at https://publons.com/publon/10.1002/brb3.1802.

## Supporting information

Supplementary Material

## Data Availability

This article and its supplementary files include all data generated or analyzed during the presented study.
