# Supplementary material for: Collagen‐rich deposit formation in the sciatic nerve after injury and surgical repair: A study of collagen‐producing cells in a rabbit model
Source: Brain Behav. 2020 Aug 15;10(10):e01802. doi: 10.1002/brb3.1802 (PMC7559634; doi:10.1002/brb3.1802)
Supplement: Supplementary file 1 — Supplementary Material [file BRB3-10-e01802-s001.docx]

**SUPLEMENTARY MATERIALS**

**Collagen-rich deposit formation in the sciatic nerve after injury and surgical repair: a study of collagen-producing cells in a rabbit model.** Jolanta Fertala ^1^, Michael Rivlin ^1,2^, Mark L. Wang ^1,2^, Pedro K. Beredjiklian ^1,2^, Andrzej Steplewski ^1^, and Andrzej Fertala ^1*^

^1^Department of Orthopaedic Surgery, Sidney Kimmel Medical College, Thomas Jefferson University, Philadelphia, PA

^2^Rothman Institute of Orthopaedics, Thomas Jefferson University Hospital, Philadelphia, PA


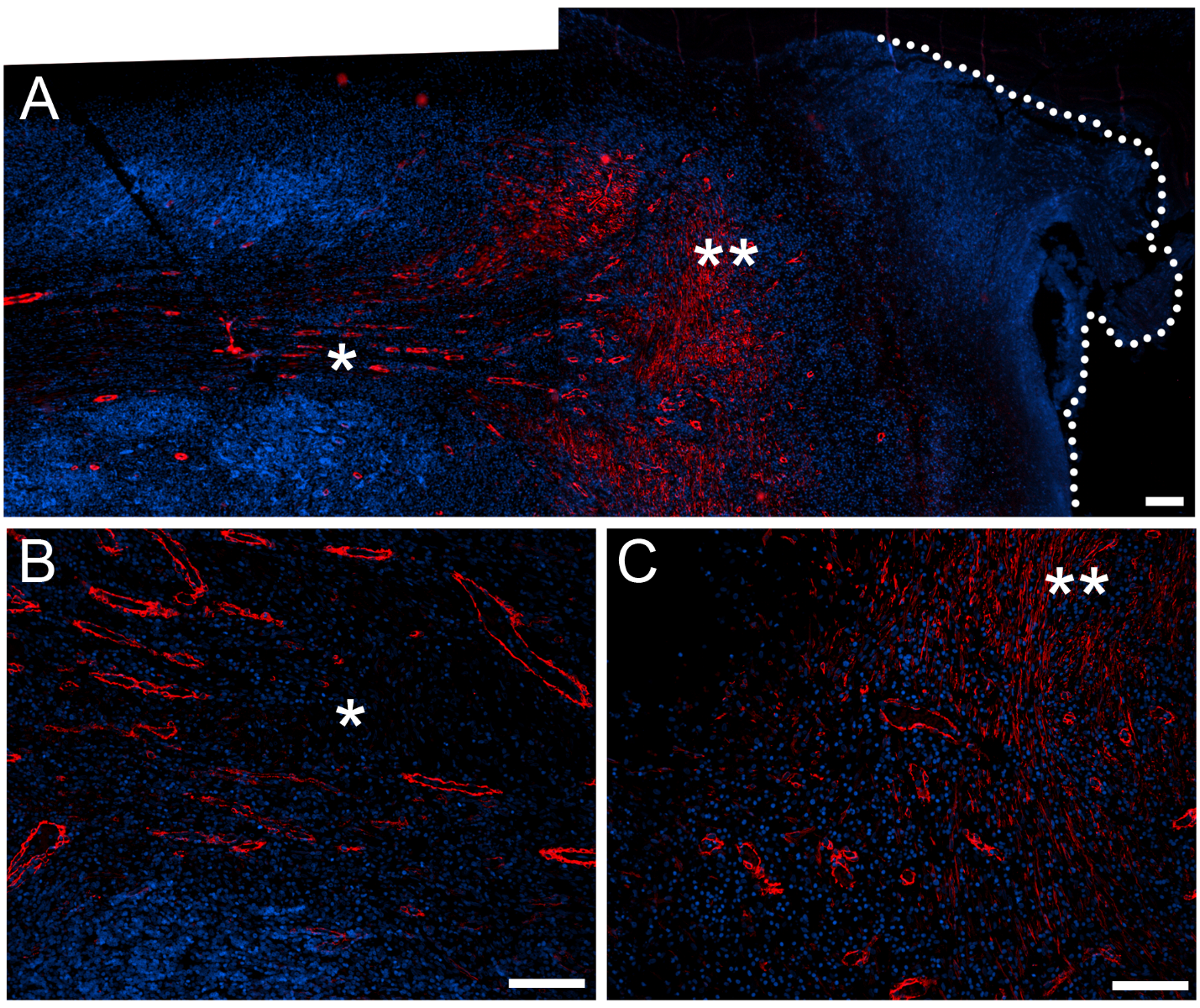


**Supplementary Figure 1**. A longitudinal section of a stump of the sciatic nerve collected 4 weeks after injury. The specimen was stained to detect αSMA present in the blood vessels formed in parallel and perpendicular orientations to the long axis of the stump. A, Low magnification of the end of a stump. B, C. High magnification of the areas indicated with asterisks. A dotted line marks the margin of the stump. In all panels, DAPI staining indicates the location of cells. Bars = 100 µm


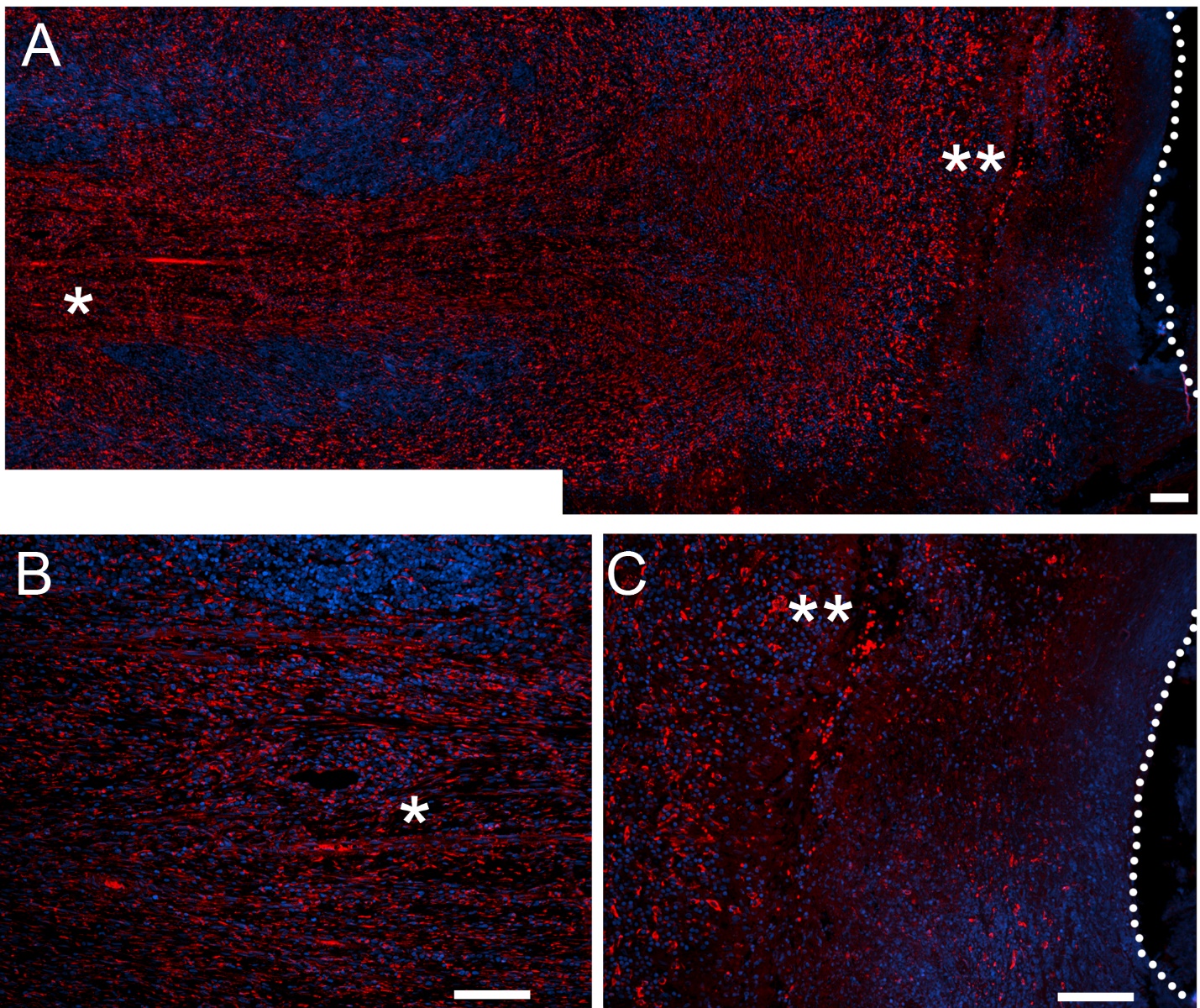


**Supplementary Figure 2**. A longitudinal section of a stump of the sciatic nerve collected 4 weeks after injury. The specimen was stained for HSP47 to depict cells that actively produce collagenous proteins. A, Low magnification of the end of a stump. B, C. High magnification of the areas indicated with asterisks. A dotted line marks the margin of the stump. In all panels, DAPI staining indicates the location of cells. Bars = 100 µm


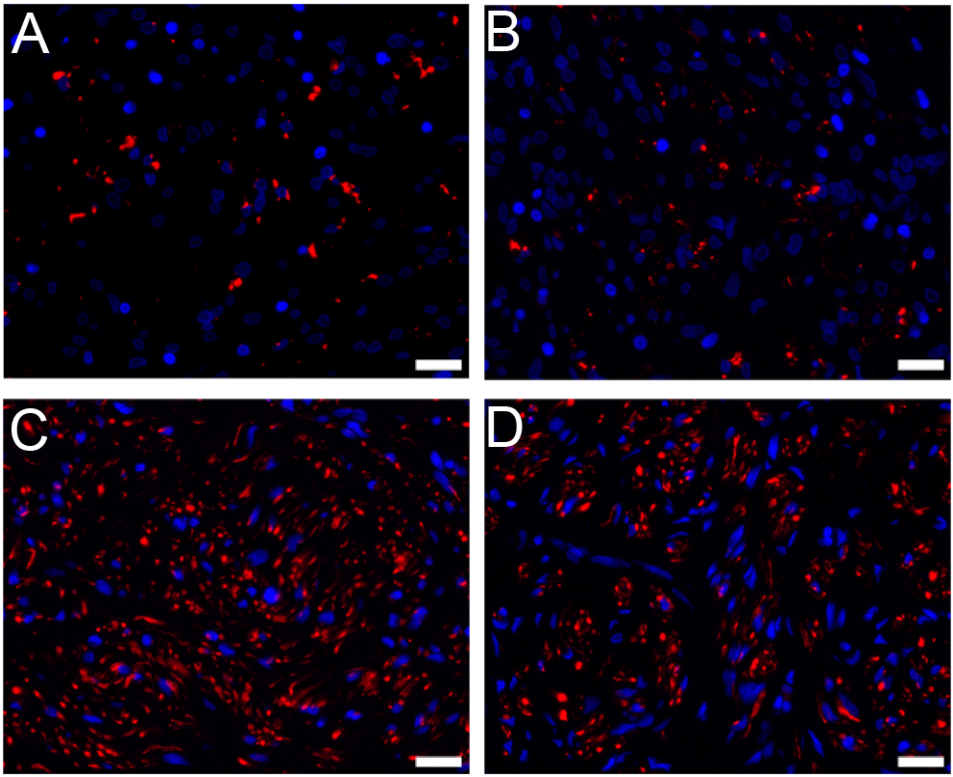


**Supplementary Figure 3**. The growth of neurofilaments in the injured sciatic nerves. The proximal (A, C) and the distal (B, D) stumps of the nerves collected 6 weeks (A, B) and 10 weeks (C, D) after injury are presented. Bars = 20 µm

| **Target** | **Primary antibody:**  **Host**  **Manufacturer**  **Catalog number**  **Dilution/Incubation** | **Secondary antibody:**  **Host**  **Manufacturer**  **Catalog number**  **Dilution/ Incubation**  **Fluorophore/Chromophore** |
| --- | --- | --- |
| HSP47 | **Mouse**  **Santa Cruz Biotechnology, Inc.**  Sc-5293  1:500/4°C/ON | Goat  LifeSciences/Thermo Fisher Scientific  A11032 and A10689  1:1000/RT/1h  Alexa Fluor 594 |
| αSMA | Mouse  Abcam and Santa Cruz **Biotechnology, Inc.**  ab-7817  PA5-18292  1:200/RT/2h  For double staining:  1:200/4°C/ON | Goat  LifeSciences/Thermo Fisher Scientific  A11032 and A10689  1:1000/RT/1h  Alexa Fluor 594 or Alexa Fluor 488 |
| Pan-axonal neurofilament marker | Mouse  BioLegend Inc  SMI-311  1:200/RT/2h | Goat  LifeSciences/Thermo Fisher Scientific  1:1000/RT/1h  Alexa Fluor 594 |

**Table 1. Antibodies and staining conditions applied in immuno-histological assays.**

**Table 2. Mean and S.D. values for all parameters measured in defined sites of the analyzed nerves.**

| **TEST** | **MARKER** | **COMPARISON** | **LOCATION** | | **Mean** | **±S.D.** |
| --- | --- | --- | --- | --- | --- | --- |
| Quantitative immuno-histology | αSMA | Px/Ds | ^a^Px | | 9.13 | 2.12 |
|  |  |  | ^a^Ds | | 2.62 | 2.10 |
|  |  | IL/EL | ^b^IL | | 5.37 | 1.17 |
|  |  |  | ^b^EL | | 6.38 | 2.46 |
|  |  | Px/Ds*IL/EL | Px | IL | 9.46 | 2.23 |
|  |  |  |  | EL | 8.80 | 3.64 |
|  |  |  | Ds | IL | 1.28 | 2.58 |
|  |  |  |  | EL | 3.96 | 3.29 |
|  |  | In/Un | ^c^In | IL | 1.20 | 0.24 |
|  |  |  | ^d^Un | IF | 0.27 | 0.30 |
|  | HSP47 | Px/Ds | ^a^Px | | 35.42 | 3.46 |
|  |  |  | ^a^Ds | | 15.65 | 2.01 |
|  |  | IL/EL | ^b^IL | | 19.91 | 3.01 |
|  |  |  | ^b^EL | | 31.20 | 2.52 |
|  |  | Px/Ds*IL/EL | Px | IL | 30.38 | 5.51 |
|  |  |  |  | EL | 40.45 | 4.17 |
|  |  |  | Ds | IL | 9.43 | 2.85 |
|  |  |  |  | EL | 21.87 | 2.85 |
|  |  | In/Un | In | IL | 15.26 | 1.90 |
|  |  |  | Un | IF | 1.09 | 2.27 |
| FTIR | ^c^AI/C | Px/Ds | ^a^Px | | 131.00 | 5.55 |
|  |  |  | ^a^Ds | | 169.51 | 6.58 |
|  |  | IL/EL | ^b^IL | | 141.34 | 5.00 |
|  |  |  | ^b^EL | | 159.14 | 7.10 |
|  |  | Px/Ds*IL/EL | Px | IL | 137.20 | 7.85 |
|  |  |  |  | EL | 124.78 | 7.85 |
|  |  |  | Ds | IL | 145.48 | 5.88 |
|  |  |  |  | EL | 193.55 | 11.78 |
|  |  | In/Un | In | IL | 142.50 | 7.44 |
|  |  |  | Un | IF | 225.56 | 11.21 |

**Legend:**

Px/Ds; Comparison of a marker measured in the proximal (Px) and the distal (Ds) stumps; for each stump results for a marker measured in the intra-luminal (IL) and the extra-luminal compartments were combined.

IL/EL; Comparison of a marker measured in the intra-luminal (IL) and the extra-luminal (EL) compartments; for each compartment results for a marker measured in the proximal (Px) and the distal (Ds) stumps were combined.

Px/Ds*IL/EL; measurements of interaction between the stump position (Px/Ds) and the luminal compartment location (IL/EL)

^a^Data combine results for the IL and EL compartments of analyzed stumps.

^b^For each luminal compartment, data combine results for both the Px and the Ds stumps.

^c^The ratio of the area of the amide I peak and the peak representing collagenous proteins in the FTIR spectra. **NOTE**: The value of the AI/C ratio is reversely proportional to the relative amount of collagen in analyzed regions.

In, injured nerve; Un, uninjured nerve; IF, intrafascicular space
